# Forming Cavities in Filling Teeth

**Published:** 1860-07

**Authors:** 


### ARTICLE VI.

### *Forming Cavities in Filling Teeth.*

To form cavities in the superior front teeth, we prefer that the upper and lower extremities of the cavity should describe an angle, the central portion being formed in any possible way the disease may indicate. By this shape and by slightly under cutting, or by drilling just within the cavity at the extreme point of the angle, a filling never can come out when properly condensed.

We have been led to express the results of our experience, by having recently read a treatise upon filling teeth, in which the author erroneously advises the obliteration of all angles in a cavity, following or agreeing with Mr. Tomes' incorrect theory of the imperfect condensation of gold in the angles of cavities. Now, so far from correct is this opinion, that we assert, it would be impossible for a good operator to fill a front approximal cavity imperfectly shaped angularly. Such a shape would enable him to insert a filling of much stronger attachment than could be obtained by an oval, round or otherwise indefinite shape.

All force applied in a cavity towards the point of its angle, instead of being expended, as Mr. Tomes says, against the walls, is directly reflected along the two planes of the angle towards the point of their junction, so that this is the first part consolidated, having received necessarily, the greatest amount of pressure during the filling of the angular portion of the cavity, and when carefully done, will always be found sufficiently solid to prohibit wedging, or at least admitting less than those whose shape approaches a circle. This also secures an entire freedom from movement of the gold whilst introducing, so that we generally aim to form a more or less decided angle at that portion of the cavity destined to receive the first layers of gold, and which is made a point d'appui for the filling.

Of course every operator experiences trouble from the rolling or rocking of the gold in large round cavities, or rather, that this is one of the most troublesome difficulties to avoid, and is too often attempted by altering the shape of the gold to be introduced. This originates entirely in the round condition of the cavity, which is frequently hard to avoid, from the extent of the disease and from the extreme sensibility of the tooth preventing an operator from forming any desired shape, and thus induced to use unusual or uncommon shape of gold to overcome to a certain extent this difficulty ; but this is never experienced when an angle is formed by the excavator or by the disease.

This holds equally good in the approximal surfaces of the molars and bicuspids, and when an angular shape cannot be obtained, the next best is a square one, to obviate the two great defects of a round cavity, that is rocking of the gold, and non-retention of the filling.

This position holds good whether gold is used in pellets, rolls or folds.

A good article of gold is quite capable of being forced into any shaped cavity, sealing it in a most perfect manner for a season, so that the great desideratum in shape is for the perfect retention of the filling; regarding convenience whilst introducing the gold, we believe that the more angular a cavity can be made at two opposite sides, the more certain and accurate will be the result; as to the condensation of the gold as far as shape is concerned, this is simply a matter of fact in all cavities and under all circumstances to a good operator. Of course referring only to the common and ordinary operations, such as Taft, and Tomes, describes.

We may be allowed to complain of these two writers wasting some pages in their books about the difficulties of filling such crown cavities as are much larger within than at their orifices, recommending the cutting away of sound dentine and enamel, to secure a nearly parallel condition of the walls; such advice would be good provided the disease has attacked such portions to be cut away, but in all crown fillings the true shape of the cavity is such as may be left, after entirely removing the decay and securing the retention of the filling. Gold can be readily condensed, let the cavity be whatever shape it may, which will retain the gold, for with a smooth edge and retaining form; a crown cavity may assume any shape, and it is an easy task to merely ordinary skill. To our mind, we require less information about simple cavities than is peculiarly demanded at present, for those that are only occasional, but complicated, and requiring great ingenuity, such as underlie a filling forming a base to a crown.

Although there is something unpleasant to a grave man in the labor of an art, to be interested in what has been maliciously termed "fancy operations," yet there is scarcely a week that we are not called upon to perform one or more of such operations, and not a day that we do not see opportunities of preserving teeth did we possess the ability to do so.

Since the great advocate of crystal gold has in a measure withdrawn, we have had no ideas of value sent out upon the building up of crowns upon healthy fangs, or of restoring the lost shape of teeth, and we have been astonished as well as disappointed in finding the entire absence of this topic in two recent works upon operative dentistry, the one ignoring the subject entirely, the other treating it so indifferently as to justify the foregoing assertion.

In our practice we have had at least fifteen cases within the last three months of the partial or entire rebuilding of the crowns of teeth, and we only give a mere outline of our efforts to supply this demand, as much to declare our own want of light, as to ask for some practical and useful information upon the operation.

In some cases we have used gold posts inserted into the fangs, having formed screws upon the end to be introduced, forcing them in by this means as firmly as possible. These posts or standards have been as large as the strength of the fangs would permit, their surfaces strongly barbed by a sharp chisel, to secure the attachment of the gold in packing. The principal difficulty has been in preparing the remaining portion of the tooth to be operated upon and the best manner of introducing the gold.

In all cases there will be a large portion of the natural crown remaining, of various shapes, according to progress of disease. The extending edges or surface should be filed down until healthy or strong enamel or dentine is reached, so that, if possible, all around and within the cavity a groove should be cut, here and there a hole drilled in the sound parts, as much at a right angle with the centre of the filling as can be secured, these holes being as large as strength of part will justify. This form will aid in retaining the gold, which of course must be made as solid as possible throughout, but especially at its base.

When any extent of surface will be exposed in masticating, the filling should be supported as much as possible by the parts or standards, which are easily inserted and give unquestionable strength and solidity to the whole, enabling the compact gold to retain its position against almost any legitimate force.

In a bicuspid, for instance, the post should form the inner cusp, and is most direct in these teeth, which have two fangs. But when they have but one, the standard has to slant towards the point of cusp desired, from the centre, as it were. In molar teeth, these standards are supported from any of the fangs, or from all, according to circumstance. Generally, an entire portion of the tooth will remain of one of its sides, this aiding in the support of the gold, the standards being placed so as to support all other surfaces.

My object is not to write a treatise or an article upon this subject, but to induce some practical information upon building parts, or whole crowns of teeth, about which our profession need instruction; perhaps more than upon any other.

At this day, a simple cavity is well filled by almost every dentist, a difficult one by the great mass, but to build up a lost portion of a tooth, there are but few who deserve to be spoken of.

Those puritans who started the cry "fancy operations," have no doubt retarded the progress of our imposing efforts in this direction, holding out the idea that such statements as have been made are fabulous accomplishments, and therefore have been entirely rejected by the uninitiated, when really we cannot but think, that by a little system, a good deal of labor, a little experience and devoted efforts, would have perfected the highest branch of our art. How much this same cry has tended to induce the use of amalgams, and is at this time encouraging the introduction of such compounds as will of necessity do an immense deal of mischief, it would be difficult to say, but in many instances I

know of several persons who since Townsend's recommendation and others, have continued to use this paste as well as other soft ingredients, equally questionable, who were quite capable of using gold in almost any desirable manner, but have since declared that they were alone suitable for operations reforming the crowns of teeth.

